# Digital Media as a Proponent for Healthy Aging in the Older Chinese American Population: Longitudinal Analysis

**DOI:** 10.2196/20321

**Published:** 2020-06-16

**Authors:** Sara Shu, Benjamin K P Woo

**Affiliations:** 1 University of California, Los Angeles Sylmar, CA United States; 2 College of Osteopathic Medicine of the Pacific Western University of Health Sciences Pomona, CA United States

**Keywords:** geriatrics, health promotion, health education, social media, Parkinson disease, fall prevention, oral health, pulmonary disease, gastrointestinal health

## Abstract

**Background:**

Ensuring health literacy among underserved populations is essential amid an aging population. Accessible and appropriate (both culturally and linguistically) information is important when considering digital media education for older Chinese Americans.

**Objective:**

This study aims to investigate how social media fare over time in disseminating health information and how we may most effectively educate this population.

**Methods:**

For this study, 5 geriatric-themed educational videos about Parkinson disease, fall prevention, gastrointestinal health, oral health, and pulmonary disease were uploaded to YouTube. Data were collected over a 40-month period. Descriptive statistics and chi-square analysis were used to compare results from the first and second 20-month periods.

**Results:**

In 40 months, the 5 videos in aggregate accrued 1171.1 hours of watch time, 7299 views, and an average view duration of 9.6 minutes. Comparing the first and second 20-month periods, there was a significant increase in mobile device usage, from 79.4% (3541/4458) to 83.3% (2367/2841). There was no significant difference in the usage of various external traffic sources and methods of sharing, with WhatsApp accounting for the majority of sharing in both 20-month periods.

**Conclusions:**

Our study provides insight into where to focus future strategies to optimize digital media content, and how to best recruit, direct, and disseminate health education to an older adult Chinese American population. Combining the success of YouTube, social media, and messaging platforms such as WhatsApp can help to transcend cultural and linguistic barriers to promote healthy aging.

## Introduction

By the year 2060, there will be roughly 98 million Americans aged over 65 years, roughly 1 in 5 people, in large part due to the aging baby boom generation [1]. With aging come inevitable challenges of chronic diseases, falls, physical activity, oral health, and mental health concerns that can largely impact quality of life. Parkinson disease, the second-most common neurodegenerative disease after Alzheimer disease, is expected to affect nearly 1.2 million Americans by 2030 [2]. Similarly, falls have become a leading cause of injury among older adults, with a projected 100,000 fatal falls per year and direct treatment costs expected to reach US $101 billion by 2030 [3]. Challenges like these result in significant financial and emotional burden for families and caregivers, emphasizing the need to optimize care for this aging population in the coming decades.

The older adult population is not only growing, but also becoming more racially and ethnically diverse, making inequities in health and access to resources more apparent [4]. Nationally implemented healthy aging initiatives, programs, and services therefore need to consider the unique needs of different subpopulations, and provide culturally and linguistically appropriate materials. Chinese Americans are one minority group that underutilize health resources; as a result, they are at risk for delayed diagnoses, and suboptimal treatment and management of a variety of chronic health conditions [5-7]. There remain cultural, educational, and linguistic barriers that present challenges in health literacy, access, and information dissemination [8,9].

Amid an aging population, distribution of health education over the internet and social media can contribute to healthy aging. Today, more and more Americans turn to the internet for health information. Social media has transformed into a platform for health communication among the general public, patients, and health professionals [10]. Among them, YouTube has become one of the world’s most popular social media platforms [11]. Digital health education dissemination holds promise in helping to bridge cultural and linguistic barriers that have previously precluded populations from access to such information. It therefore behooves us to study how populations utilize and access digital health information to tailor how best to distribute and promote health literacy among underserved populations.

Previous studies have shown that YouTube is effective in delivering dementia knowledge to older Chinese Americans [12-14]. Another study has analyzed Twitter as a health information relaying platform [15]. Furthermore, Facebook advertising has proven promising for the dissemination of dementia and hypertension information [16,17]. In addition, more recent studies have suggested a rise in WhatsApp use among older Chinese Americans in sharing dementia education [18,19]. However, few studies have investigated other health education topics (even in aggregate) and the role of social media in their dissemination to this population. In this paper, we aim to determine the efficacy of YouTube as a medium for delivering a variety of aging-related health education resources, and study the change in modes of viewing and sharing across different social media platforms over time. To our knowledge, this is the first longitudinal study of 5 different geriatric-themed videos in the older Chinese American population.

## Methods

### YouTube

A board-certified psychiatrist delivered 5 geriatric-themed educational talk shows in Cantonese at the radio station KMRB AM1430 in Los Angeles. Real-time recordings were then individually uploaded to YouTube. Average video length was 36.4 minutes. Topics addressed include Parkinson disease, fall prevention, gastrointestinal health, oral health, and pulmonary disease.

### Sample

The sample of this study included YouTube video viewers over a 40-month period (November 2016 to March 2020).

### Statistical Analysis

Data were extrapolated from YouTube Analytics. Parameters recorded included number of views, watch time, average view duration, devices used to view, traffic sources, and modes and means of sharing via various social media platforms. The first and second 20-month intervals were dichotomized (November 2016 to July 2018, and July 2018 to March 2020). Descriptive statistics and chi square test were used to compare data collected between the first and second 40-month intervals.

This study used anonymous data exclusively collected by YouTube. A waiver for Institutional Review Board exemption was obtained through the Human Subjects Protection Committee of University of California, Los Angeles.

## Results

In 40 months, the 5 videos in aggregate accrued a total of 1171.1 hours of watch time and 7299 views, and an average view duration of 9.6 minutes. A breakdown of each of the 5 video topics is shown in Table 1. Data were then dichotomized into two 20-month intervals: November 2016 to July 2018, and July 2018 to March 2020. Between November 2016 and July 2018, the recorded YouTube videos accrued a total of 738.6 hours of watch time and 4458 views, and an average view duration of 9.9 minutes. Between July 2018 and March 2020, there were 432.5 hours of watch time and 2841 views, and the average view duration was 9.1 minutes. Overall, the latter 20 months had a decrease in total watch time (738.6 versus 432.5 hours), the number of views (4458 versus 2841 views), and the average view duration (9.9 versus 9.1 minutes).

**Table 1:** Statistics of 5 videos over 40 months.

Video topics	Number of views	Hours watched	Average view duration (minutes)	Total video length (minutes)
Parkinson disease	3092	501.5	9.7	38
Falls	1990	352.6	10.6	37
Constipation and diarrhea	1076	112.4	6.3	27.5
Oral health	636	107.7	10.2	39
Pulmonary disease	505	96.9	11.5	40.5
Total	7299	1171.1	9.6	182

Average view duration on computers increased 13.0% from 8.4 to 9.5 minutes between the first and second 20-month periods. In comparison, average view duration on mobile devices (mobile phones and tablets) decreased 6.9% from 10.9 to 10.2 minutes. Despite an overall decrease in views in the second 20-month period, the relative usage of computers decreased from 20.6% (917/4458) to 16.7% (474/2841), while relative mobile usage increased from 79.4% (3541/4458) to 83.3% (2367/2841). The increase in relative mobile device usage compared to computer usage from the first to the second 20-month period is statistically significant (79.4% versus 83.3%, *χ*^2^_1_=17.0, *P*<.001; Table 2).

**Table 2:** Devices used for viewing videos.

Devices	November 2016 to July 2018			July 2018 to March 2020		
	Number of views	Hours watched	Average view duration (minutes)	Number of views	Hours watched	Average view duration (minutes)
Computer	917	129	8.4	474	74.7	9.5
Mobile device	3541	609.6	10.9	2367	357.8	10.2
Total	4458	738.6	9.9	2841	432.5	9.1

Looking at traffic sources, externally sourced views remained the same between the two 20-month periods: 6.9% (308/4458) and 6.9% (198/2841) of views, respectively. Of external sources, the top platforms were Facebook, Google search, and WhatsApp. Over 40 months, traffic generated from Facebook drastically decreased from 1.8% to 0% of total views (82/4458 versus 0/2841), Google search increased from 0.9% to 1.4% of total views (41/4458 versus 39/2841), and WhatsApp decreased from 0.8% to 0.5% of total views (34/4458 versus 15/2841). Despite the significant drop in Facebook-generated traffic, there was no significant difference in traffic generated through Google search (0.9% versus 1.4%, *χ*^2^_1_=3.7, *P*=.06) or WhatsApp (0.8% versus 0.5%, *χ*^2^_1_=1.7, *P*=.19) between the two 20-month periods (Table 3).

**Table 3:** Traffic sources.

Traffic sources		November 2016 to July 2018						July 2018 to March 2020					
		Number of views		Hours watched		Average view duration (minutes)		Number of views		Hours watched		Average view duration (minutes)	
**Top 4 traffic sources**													
	Suggested videos		2024		375.9		11.1		917		161.4		10.6
	YouTube search		945		102.7		6.5		790		98.4		7.5
	Browse features		731		149		12.2		715		118.4		9.9
	External		308		34.4		6.7		198		22.4		6.8
**Top 4 external traffic sources**													
	Facebook		82		7		5.1		0		0		0
	Google search		41		2.6		3.8		39		5.3		8.1
	WhatsApp		34		6.4		11.3		15		0		12
	Other		6		1.3		13		6		0.3		3

In the first 20 months, the videos were shared 107 times through various sharing services. In the second 20 months, the videos were shared 56 times, and WhatsApp was the most utilized sharing method. However, comparing the two time periods, there is no significant difference in the usage of WhatsApp for sharing (54.2% versus 58.9%, *χ*^2^_1_=0.3, *P*=.56; Table 4).

**Table 4:** Use of sharing services during both study periods.

Sharing services	November 2016 to July 2018 (N=107)	July 2018 to March 2020 (N=56)
	Shares, n (%)	Shares, n (%)
WhatsApp	58 (54.2)	33 (58.9)
SMS text messaging	7 (6.6)	1 (1.8)
Email	5 (4.7)	4 (7.1)
Facebook	1 (0.9)	1 (1.8)
Other	36 (33.6)	17 (30.4)

## Discussion

### Principal Findings

The majority of older adults have multiple chronic conditions [20]. Rather than focusing on a single condition or disease, this study is the first to analyze in aggregate the performance of 5 aging-relevant educational videos over 40 months. By investigating how older Chinese Americans are utilizing different platforms for viewing and sharing videos, we gain valuable insight into how we may tailor future health education dissemination to this population. Overall, the latter 20-month period had a decrease in total watch time (738.6 versus 432.5 hours), the number of views (4458 versus 2841 views), and average view duration (9.9 versus 9.1 minutes). A previous study determined that 6 minutes is the average engagement time for online educational videos of varying lengths [21]. Average view duration over the 40-month period was 9.6 minutes, demonstrating that these videos succeeded in maintaining viewers’ attention and engagement.

Chi-square analysis revealed that mobile devices remain the top device used for viewing these educational videos; indeed, they experienced a statistically significant increase in usage from 79.4% (3541/4458) to 83.3% (2367/2841) over the 40 months, while computer usage decreased from 20.6% (917/4458) to 16.7% (474/2841). As there is a continued shift from computer to mobile device usage, ensuring mobile device compatibility in future digital health communication should be a priority. Furthermore, it becomes important to examine and understand any barriers and challenges that this population faces to better shape the design of future platforms and systems of health-related communication via mobile device. Barriers and challenges may include functional limitations such as visual or motor impairment, having low technology literacy, or being adverse to new methods. Work can be done to develop more user-friendly interfaces to maximize potential use among older adults. The development of user-friendly interfaces is not limited to only mobile device but extends to the development of future technologies such as voice-activated speaker devices, and the growing number of products on e-commerce platforms tailored for an aging population [22,23].

With regards to external traffic sources, while there was a significant decrease in Facebook-generated traffic, there was no significant change in Google search and WhatsApp traffic. Our study reveals an increase in Google search from 0.9% to 1.4% of total views (41/4458 versus 39/2841) and a decrease in WhatsApp from 0.8% to 0.5% of total views (34/4458 versus 15/2841). Although WhatsApp remains the top sharing service, there was no significant change in the amount of sharing that occurred between the two periods. Taken together, this study entertains questions of how to increase visibility via Google search amid a saturating field, and how to promote viewer sharing via WhatsApp. For example, future studies can investigate whether using long tail keywords (more specific keyword phrases) increase visibility via Google search.

It has previously been shown that digital recruitment via Facebook and, more recently, Instagram, is promising in directing individuals to health education materials [16,17,24]. However, the effects last only as long as the recruitment period. If strong and effective advertising is conducted at the beginning of a particular study period, the media in question could potentially experience longer-lasting visibility across future searches via Google. Work therefore needs to be done to devise novel or stronger methods of advertising (eg, Facebook, Instagram, or equivalent), and to publicize the availability of these resources to ensure enduring visibility and impact for years to come.

Finally, previous studies have shown that WhatsApp has become the preferred means of sharing dementia knowledge and is used more for its sharing capability than for its viewing function [18,19]. Although our data only provide a 40-month window into the performance of 5 different aging-themed educational videos, our study acknowledges WhatsApp’s potential to become a successful platform for disseminating information for healthy aging to the older Chinese American population. As not only a social media platform, but also a personal messaging system, WhatsApp has the potential to reach a wide audience. With so many resources now available for the internet searcher, for any single resource to have a significant impact, the methods of dissemination and incentives for viewers to share need to evolve. Future studies could investigate the strategic placement of reminders to, for example, “share via WhatsApp if you found this useful.” Other studies could incorporate the use of visual WhatsApp icons (specifically, the WhatsApp share button) to prompt and facilitate sharing via WhatsApp.

### Limitations

There are several limitations in this study. As the videos used in this study were filmed in Cantonese, the audience was limited to those in the Chinese American population who are fluent in Cantonese. Furthermore, each video retained a rather short average viewing duration of 9.9 to 9.1 minutes, which is a fraction of the average video length of 36.4 minutes. Future studies should consider shortening video lengths and incorporating more interactive elements to increase audience engagement time with the goal of improved audience experience and greater retention of educational information. As a retrospective longitudinal analysis, data collected were limited to that collected by YouTube Analytics. Being able to design a prospective study would enable us to focus on WhatsApp as a sharing service, or the effectiveness of various recruitment methods.

### Conclusions

The internet and usage of social media are continually evolving and changing the way in which we communicate health information among individuals and the medical community. YouTube is a promising and valuable tool to deliver culturally and linguistically appropriate health education to isolated populations. More studies need to be done to harness technologies now available on mobile devices with meaningful improvement in the health of older adults. In addition, future studies could investigate how WhatsApp can achieve its full potential as a top platform for health information dissemination. As such, further studies looking at both short- and long-term strategies and outcomes are necessary to learn how different populations of interest search for and disseminate information to best be able to serve and deliver pertinent health education, ensure healthy aging, and promote healthy outcomes.
